# Emergence of an echovirus 17 subtype causing a viral meningitis outbreak, Israel, 2025 to 2026

**DOI:** 10.2807/1560-7917.ES.2026.31.30.2600600

**Published:** 2026-07-30

**Authors:** Neta S Zuckerman, Eyal Nadir, Rivka Rich, Eli Somekh, Mohamed Asi, Oshrat Ayalon, Orli Megged, Talya Finn, Or Kriger, Michal Paret, Efrat Dresner, Tatyana Ruderman, Erez S Garty, Hilla De-Leon, Rinat Vasserman, Leah Weiss, Reut Gabay, Ilana S Fratty, Yaniv Lustig, Danit Sofer, Merav Weil

**Affiliations:** 1 Central Virology Laboratory, Public Health Services, Ministry of Health, Chaim Sheba Medical Center, Ramat Gan, Israel; 2 School of Public Health, Gray Faculty of Medical and Health Sciences, Tel Aviv University, Tel Aviv, Israel; 3 Division of Epidemiology, Ministry of Health, Jerusalem, Israel; 4 Department of Pediatrics, Mayaney Hayeshuah Medical Center, Bnei Brak, Israel; 5 The Clinical Microbiology Laboratory, Shaare Zedek Medical Center, Jerusalem, Israel; 6 Faculty of Medicine, Hebrew University of Jerusalem, Jerusalem, Israel; 7 Pediatric Department, Shaare Zedek Medical Center, Jerusalem, Israel; 8 Department of Infectious Diseases, Sanz Medical Center, Netanya, Israel; 9 Faculty of Medicine, Ariel University, Ariel, Israel; 10 Public Health Services, Ministry of Health, Jerusalem, Israel; 11 Haifa University, Faculty of Medicine, Haifa, Israel; 12 Pediatric Infectious Diseases Unit, Sheba Medical Center, Ramat Gan, Israel; 13 Gray Faculty of Medical and Health Sciences, Tel Aviv University, Tel Aviv, Israel; 14 Infectious Diseases Unit, Sheba Medical Center, Ramat Gan, Israel; 15 Health Intelligence Agency, Ministry of Health, Jerusalem, Israel; 16 The Israel Center for Disease Control, Israel Ministry of Health, Ramat Gan, Israel

**Keywords:** echovirus 17, meningitis, surveillance, clinical, sequencing

## Abstract

An outbreak of enteroviral meningitis occurred in Israel between August 2025 and May 2026. Echovirus type 17 was identified in 72 hospitalised patients, most of them infants (n = 44). Many patients (n = 45) were from densely populated Haredi (strictly Orthodox) communities. Clinical course was mild. Phylogenetic analysis suggests extensive local transmission. Echovirus 17 infections declined abruptly following the implementation of community restrictions imposed during a military conflict. This outbreak highlights the value of routine molecular enterovirus surveillance.

Echovirus 17 (E17), belonging to the *Enterovirus betacoxsackie* species, is not a common cause of enteroviral meningitis [1] but has previously been associated with outbreaks, neurological complications and fatalities [2-4]. Between August 2025 and May 2026, patients with E17 meningitis were identified in Israel through the national enterovirus surveillance programme. Here we describe the epidemiological, clinical and molecular characteristics of the outbreak and investigate its transmission dynamics using national surveillance and phylogenetic analysis.

## Multicentre detection of the echovirus 17 outbreak

The National Reference Centre for poliovirus and enteroviruses at Israel’s Ministry of Health has maintained molecular surveillance for non-polio enteroviruses over the past decade, despite these viruses not being notifiable. Surveillance is based on enterovirus typing of cerebrospinal fluid (CSF) specimens from patients with viral meningitis at Sheba Medical Center, Israel’s largest tertiary hospital which serves a socio-demographically diverse population representative of Israel's major ethnic and religious communities in central Israel. This surveillance has enabled the identification and monitoring of seasonal outbreaks caused by multiple enterovirus subtypes, most commonly E18, E5, E6, E30, coxsackievirus B5 (CVB5), E4 and CVB2 [5].

**Figure 1:**
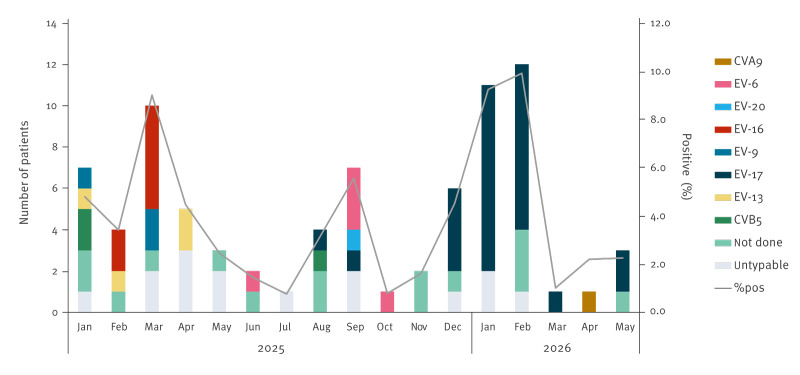
Timeline of detection of enterovirus types in cerebrospinal fluid samples, Sheba Medical Center, January 2025–May 2026 (n = 26)

CVA: Coxsackie A virus; CVB: Coxsackie B virus; EV: echovirus.

Through this established surveillance framework, E17 was detected in Israel for the first time in a hospitalised patient with meningitis in August 2025. This was followed by a marked increase in patients with enterovirus-associated meningitis beginning in December 2025, with numbers peaking in January and February 2026 (Figure 1). In total, 26 E17-positive patients were identified at Sheba Medical Center between August 2025 and May 2026. During the peak months of early 2026, additional hospitals alerted the Ministry of Health about an atypical, synchronous increase in viral meningitis admissions testing positive for enteroviruses. These hospitals are in various geographical regions in Israel and include Mayanei Hayeshua Medical Center in Bnei Brak in central Israel, which serves a large Haredi (strictly Orthodox) community, Shaare Zedek Medical Center in Jerusalem, which serves a sizable Haredi population, and Laniado Medical Center in Netanya, which serves a more heterogeneous population.

To characterise the emerging subtype, 52 enterovirus-positive CSF specimens from the additional reporting hospitals were selected with simple random sampling during the peak outbreak months (January–February 2026) and submitted to the National Reference Centre for enterovirus typing. Of these 52 specimens 46 were laboratory-confirmed as E17 using Sanger sequencing [6]. In total, 72 patients with laboratory-confirmed E17 viral meningitis were identified during the outbreak period within the surveillance at Sheba Medical Center (n = 26) and the additional hospitals (n = 46).

## Patient characteristics and clinical picture

Demographically, the outbreak occurred predominantly among the Jewish ethnic group, with one patient identified in the Arab ethnic group (Table). Most patients (45/72; 62.5%) belonged to the Haredi community and two (2.8%) were non-Haredi. For 25 (34.7%) patients, information about the community was not known. Geographically, most of these patients were from municipalities characterised by high population density within the Haredi communities, primarily Bnei Brak (n = 19), Jerusalem (n = 12), Netanya (n = 11), Modi'in Illit (n = 5) and Beit Shemesh (n = 4). The outbreak predominantly affected paediatric patients: 44 (61.1%) were infants (aged < 1 year) and 15 (20.8%) were children and teenagers (aged 1–17 years). Thirteen (18.1%) patients were aged 18–40 years. Overall, the clinical course was mild for viral meningitis — with no major neurological complications or fatalities and all patients eventually recovered. Fever was the predominant clinical feature, recorded in 88.9% (64/72) of patients (Table). Clinical presentation varied by age; toddlers and infants presented with fever alongside at least one of the following signs: vomiting, a bulging fontanelle, restlessness, rash, drowsiness or respiratory symptoms. Adults presented with fever accompanied by a severe headache, vomiting or neck stiffness (Table).

**Table:** Demographic, epidemiological and clinical characteristics of patients with laboratory-confirmed echovirus 17 viral meningitis, Israel, August 2025–May 2026 (n = 72)

Characteristics	Patients	
	n	%
Age group		
Infants (< 1 year)	44	61.1
Children and teenagers (1–17 years)	15	20.8
Adults (18–40 years)	13	18.1
Ethnic group		
Jewish	71	98.6
Arab	1	1.4
Community		
Haredi	45	62.5
Non-Haredi	2	2.8
Unknown	25	34.7
Admitting hospital		
Sheba	26	36.1
Mayanei HaYeshua	18	25.0
Shaare Zedek	17	23.6
Laniado	11	15.3
Clinical presentation		
Fever	64	88.9
Vomiting	24	33.3
Headache	24	33.3
Drowsiness	10	13.9
Respiratory symptoms	7	9.7
Photophobia	6	8.3
Restlessness	5	6.9
Rash	7	9.7
Neck stiffness	5	6.9
Bulging fontanelle	3	4.2
Runny nose	4	5.6
Petechiae	1	1.4
Duration of fever (days)		
Median	2	
Range	1–7	
Duration of hospitalisation (days)		
Median	3	
Range	1–7	

## Phylogenetic analysis and transmission dynamics

**Figure 2:**
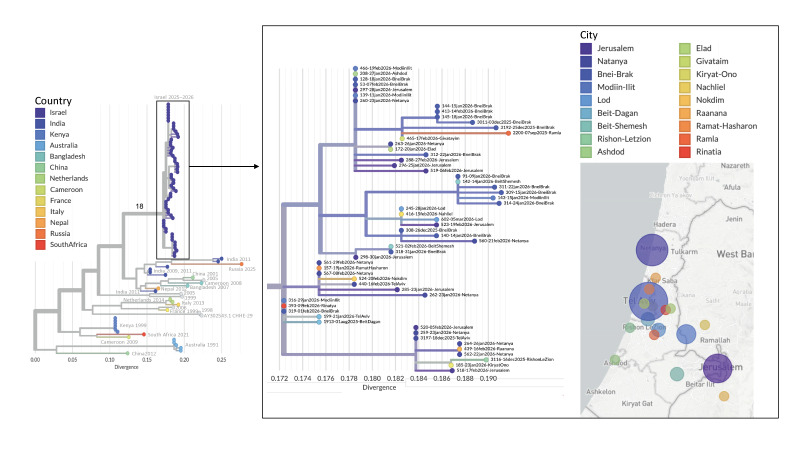
Phylogenetic analysis of echovirus 17 sequences from Israel (n = 57) and globally (n = 36), 1991–2026^a^

Phylogenetic analysis was performed using the ca 300 nt viral protein 1 (VP1) gene fragment obtained by Sanger sequencing for enterovirus typing [6]. Of the 72 E17-positive samples, 57 yielded sequences of sufficient quality and size for phylogenetic analysis (GenBank accession numbers: PZ673799–PZ673855) and were analysed together with 36 global publicly available E17 sequences. Phylogenetic analysis showed that all Israeli outbreak sequences formed a distinct cluster that was genetically distant from the global E17 sequences, differing by 18 nt across the analysed VP1 fragment. Notably, the extensive branching within the Israeli cluster suggests that the patients represented only a fraction of the circulating viral population, consistent with undetected community transmission which may be from non-hospitalised infections and cases managed at hospitals not reporting to the surveillance network. Geographically, the phylogenetic analysis did not reveal city-specific clusters. Instead, sequences from different municipalities were interspersed throughout the phylogeny, consistent with frequent transmission and extensive mixing between cities across Israel (Figure 2).

E: echovirus.

^a^ Sequences of sufficient quality were obtained from 57 of the 72 patient samples.

Phylogenetic analysis based on partial VP1 sequencing (300 nt) of E17 obtained from patients during the 2025–2026 outbreak in Israel and publicly available global E17 sequences retrieved from the NCBI GenBank database (https://www.ncbi.nlm.nih.gov/genbank/). The left panel shows the complete phylogeny, with sequences coloured by the country of origin. The Israeli cluster is enlarged in the middle panel, and the right panel shows the geographical distribution of the Israeli cases. Sequences and map are coloured by the patient municipality of residence (city). Numbers on branches indicate the inferred number of nt substitutions, and branch lengths are proportional to genetic divergence. Phylogenetic analysis was performed in Nextstrain using the GTR substitution model [14].

The designations employed and the presentation of material on this map do not imply the expression of any opinion whatsoever on the part of the European Union concerning the legal status of any country, territory, city or area or of its authorities, or concerning the delimitation of its frontiers or boundaries. Sampling locations are displayed according to collection records. Geographical designations are used solely for epidemiological analysis and do not imply any position regarding legal status or sovereignty.

## Discussion

Echovirus 17 is a rare type that has not caused major outbreaks in recent years. Thus, this surge between December 2025 and February 2026 was unexpected both in its scale and its winter occurrence, as enteroviruses usually peak in summer [7]. However, this off-season activity aligns with historical data from Israel (2016–2024) showing that certain types can circulate during colder months [5], a pattern that might also have been influenced by regional warming trends and increasingly mild winters [8-11]. More importantly, the unusual timing of the outbreak may reflect the lack of pre-existing immunity to a newly emerging enterovirus type. The outbreak declined abruptly in late February 2026, with the number of cases dropping from a peak of 12 in February to one in March. This abrupt cessation of transmission temporally coincided with a local military conflict and the subsequent implementation of stringent nationwide restrictions on public gatherings by the Israeli government (Home Front Command), which remained in effect from 28 February to mid-April 2026. Since transmission was concentrated mainly in high-density areas characterised by frequent communal interactions, strict regulations on social gatherings and school closures likely played a key role in disrupting viral transmission chains. A similar phenomenon was observed during the COVID-19 pandemic when enterovirus incidence significantly declined during periods of stringent community restrictions and lockdowns [10,12]. No outbreak-specific public health interventions were implemented, as transmission declined rapidly before such measures were considered necessary.

Although the outbreak described here was confined to Israel, it highlights the importance of maintaining molecular surveillance including in highly interconnected populations. These communities in Israel have close familial, social and religious ties with similar communities abroad, resulting in frequent international travel and opportunities for the cross-border movement of pathogens. The public health relevance of these transnational networks has previously been demonstrated during the vaccine-derived poliovirus type 2 (cVDPV2) outbreak in 2022, which linked transmission chains across environmental and acute flaccid paralysis cases in Israel, the United Kingdom and the United States [13]. As non-polio enteroviruses share the same faecal-oral and respiratory transmission routes as poliovirus, continued molecular surveillance is important for the early detection and monitoring of emerging enteroviruses.

This study has several limitations. Because non-polio enteroviruses are not notifiable in Israel, nationwide case ascertainment relies on the national surveillance system and referrals from some hospitals and may have not captured all cases occurring across the country. In addition, phylogenetic analyses were based on partial VP1 sequences rather than whole genomes, limiting genomic resolution. The surveillance system captures only hospitalised patients with meningitis and therefore does not reflect the full spectrum of E17 infections in the community. Finally, epidemiological metadata were incomplete for some patients, limiting more detailed analyses of transmission patterns and population characteristics.

## Conclusion

From a public health perspective, this outbreak of enteroviral meningitis underscores the need for clinicians to maintain a high index of suspicion for enteroviral infection, even outside the typical enterovirus season. Furthermore, because this specific strain circulated within a globally connected sub-population, these findings highlight the importance of routine molecular enterovirus typing as a core component of public health surveillance. Implementing such surveillance is essential not only for identifying domestic cryptic transmission chains but also for uncovering transnational epidemiological links, enabling early identification of emerging variants and supporting timely public health responses through international reference networks.

## Data Availability

Sequences have been deposited in GenBank under accession numbers PZ673799–PZ673855.
